# Exploring residents and supervisors’ workplace learning needs during postgraduate medical education

**DOI:** 10.5116/ijme.6470.d9ed

**Published:** 2023-05-31

**Authors:** Marieke Robbrecht, Myriam Van Winckel, Koen Norga, Mieke Embo

**Affiliations:** 1 Ghent University, Faculty of Medicine and Health Sciences, Department of Internal Medicine and Pediatrics, Ghent, Belgium; 2 Antwerp University, Faculty of Medicine and Health Sciences, Wilrijk, Belgium; 3 Ghent University, Faculty of Psychology and Educational Sciences, Department of Educational Studies, Ghent, Belgium

**Keywords:** Postgraduate medical education, workplace learning, qualitative research

## Abstract

**Objectives:**

To identify the main enablers and challenges for workplace learning during postgraduate medical education among residents and their supervisors involved in training hospital specialists across different medical specialties and clinical teaching departments.

**Methods:**

A qualitative explorative study using semi-structured focus group interviews was employed. A purposeful sampling method was utilized to invite participants who were involved in postgraduate medical education for hospital specialist medicine at two universities. Hospital physicians in training, also called residents (n=876) and supervisors (n=66), were invited by email to participate. Three focus groups were organized: two with residents and one with supervisors. Due to the COVID-19 pandemic rules prohibiting real group meetings, these focus groups were online and asynchronous. The data was analyzed following an inductive thematic analysis.

**Results:**

The following overarching themes were identified: 1) the dual learning path, which balances working in the hospital and formal courses, 2) feedback, where quality, quantity, and frequency are discussed, and 3) learning support, including residents’ self-directed learning, supervisors’ guidance, and ePortfolio support.

**Conclusions:**

Different enablers and challenges for postgraduate medical education were identified. These results can guide all stakeholders involved with workplace learning to develop a better understanding of how workplace learning can be optimized to improve the postgraduate medical education experience. Future studies could focus on confirming the results of this study in a broader, perhaps international setting and exploring strategies for aligning residencies to improve quality.

## Introduction

Postgraduate medical education (PGME) plays a critical role in the development of medical specialists, equipping them with the knowledge and skills necessary to provide high-quality patient care. Traditionally, PGME is based on workplace learning (WPL), which provides a hands-on approach to learning and assessment during real-life patient care.^1^^-^^4^  It is an effective way for medical residents to acquire the necessary competencies to function as independent physicians.

The use of WPL has evolved throughout the last decades in response to changes in medical and technological advancements, as well as societal and political demands for proof of competency.^5^^, ^^6^ This shift in focus has led scientific research to explore the learning processes that occur in the clinical workplace.^7^ As a result, there has been an increase in the literature examining the enablers and challenges of workplace learning practices in clinical settings.^1^^,^^2^^, ^^8^^-^^12^

The majority of literature on residents’ and supervisors’ experiences has focused on specific educational topics, such as workplace-based assessment and feedback, or disciplines, such as internal or surgical medicine.^13^^-^^16^ However, there is little research on the overarching features of WPL practices across various medical specialties and clinical learning departments. Therefore, it is valuable to investigate the use of WPL programs. This will help to identify factors that contribute to their success.

This study is part of a larger multidisciplinary research project. We conducted a qualitative study that aimed to understand the current needs of residents and supervisors during WPL. The research question of this study is to identify the main enablers and challenges of residents and their supervisors, involved in the training of hospital specialists across different medical specialties and clinical teaching departments.

## Methods

### Study setting

This study was carried out in the context of PGME for hospital specialist medicine in Flanders, Belgium. Medical students who have completed the six-year undergraduate and graduate medical curriculum (Bachelor’s and Master’s degrees) are eligible to apply directly for a hospital specialist medical training curriculum that lasts for four to six years.

The hospital specialist training curriculum consists of two parts. The first part is a WPL curriculum that takes place at different clinical training sites accredited for the specialty. The candidates for hospital specialist training, also known as residents, usually rotate every three to twelve months within and between different hospitals in and outside of Belgium. As employees of the hospital, they are expected to provide clinical care and are mentored by staff physicians who act as their supervisors. Many hospitals provide training to residents from different universities. An ePortfolio tool is used to support the learning process and evaluate their learning progress.

The second part of the curriculum is the Master of Specialist Medicine (MSM) curriculum, which is completed at the residents’ university. This curriculum consists of theoretical courses, practical training, assessment, and a master’s thesis. While the MSM curriculum is separate from the workplace curriculum, it is organized during the same years. Clinical evaluations by supervisors and annual reports from the input in the ePortfolio, together with successful completion of the Masters’ degree, are evaluated by a certification committee that advises the Flemish authorities to issue the license to practice as a medical specialist at the end of training.

### Study design and participants

A qualitative exploratory study was conducted using focus group interviews to thoroughly explore the needs of residents and supervisors regarding WPL.^17^ A purposeful sampling method was utilized to invite participants who were involved in PGME for hospital specialist medicine. The study was conducted at Ghent University and Antwerp University. In April 2020, residents (n=876) and supervisors (n=66) were invited to participate by email. Participants who responded positively (residents n=65, supervisors n=13) were provided with a link for registration to the online focus group tool. Three focus groups were conducted with registered participants: one with supervisors (n=9) and two with residents (n=14, n=19). All groups consisted of mixed genders and participants with various years of experience. The group of residents consisted of surgical (n=10), internal (n=16), and other (n=6) disciplines. For one resident there was no demographic data available. The group of supervisors consisted of clinicians from surgical (n=3), internal (n=3), and supportive (n=2) disciplines. Also, one curriculum manager participated. To encourage participants to express their thoughts and perceptions freely, they were given the option to anonymize themselves using nicknames. Approval from the Ethical Committee of Ghent University (BC-07808) was obtained, and all registered participants provided written informed consent prior to the focus group sessions.

### Focus groups

Focus groups were organized to gain insight into participants’ thoughts and to allow interaction between participants. Due to the COVID-19 pandemic, physical group meetings were not feasible; therefore, online asynchronous focus groups were organized instead. Separate focus groups were conducted for residents and supervisors to allow participants to express their opinions freely among peers and to identify any differences in perceptions between the two groups.^18^

### Data collection

Data for the study were collected from May to July 2020 using the online tool FocusGroupIT. The focus groups were moderated by researchers affiliated with the SBO Scaffold project (AA, MR, SVO) with backgrounds in communication sciences, medicine, and educational sciences. The focus groups discussed topics such as the course of a working day, positive and negative experiences regarding WPL, feedback and evaluation, training, and learning goals.

For each focus group, a two-week period was allocated during which a new topic was posted every three days, accompanied by three to four questions. Participants were able to respond electronically at any time convenient to them. The responses were in free text format and visible to all participants, allowing them to read each other's contributions. The moderators encouraged interaction and introduced clarifying questions when appropriate. Participants were notified via email when a new topic or comment was posted. After each focus group, an excerpt of the responses was downloaded in PDF format. The researchers used saturation of answers as a criterion to decide when no further recruitment was necessary.

### Data analysis

An inductive approach to thematic analysis was employed using NVivo 12. The analysis involved six phases.^19^ The first author (MR) conducted all six phases. To ensure triangulation of coders, two other researchers (ME, HD) conducted independent parallel coding in phases one to four. In the first phase, the researchers became familiar with the data by reading and highlighting meaningful data. The second phase consisted of generating initial codes that were meaningful to the research question. In the third phase, initial codes that were found to be similar were grouped to identify recurring themes. The fourth phase comprised reviewing the themes. In the fifth phase, themes were clustered and defined into themes and sub-themes. In the final phase, the report was written.

## Results

Within our analysis, we were able to retain three themes clustering eight sub-themes. The most relevant information about each theme is presented below. Table 1 summarizes the main findings of this explorative study, explaining them in more detail in what follows.

### Dual learning path

As described in the study context, residents follow a curriculum consisting of two parts at the same time. They complete their training both on the job during clinical practice (WPL) and during formal courses and informal training moments (MSM). Perceived enablers and challenges are described within this overarching theme.

#### Workplace learning

Most residents perceived being actively involved in performing various aspects of the job as most valuable for their learning, such as direct patient contact, discussions with supervisors, group discussions on patients, interaction with peers, and presentations on clinical cases. Additionally, residents highlighted that direct observation by experienced professionals and gradually gaining increasing independence in a safe learning environment were key factors for a positive WPL experience.

"I love this!!! So on the one hand, this gives me all the independence I want, but on the other hand, I always do this in a safe learning environment where I get lots of feedback daily!" (Resident1, medical specialty)

While residents reported positive learning experiences, they also identified several areas for improvement, with the most common concern being the inadequate provision of protected educational time for learning and supervision within the workplace. According to the residents, there was an imbalance between work and learning, which was attributed to either the demands of the clinical department or the significant amount of time spent on administrative tasks related to patient care that did not contribute effectively to their clinical competency development. Supervisors were willing to provide more guidance but were unable to do so because of time constraints, which many residents acknowledged. Conversely, one supervisor suggested that some residents purposefully did not engage with their clinical development and hid in the clinical workflow. Both supervisors and residents expressed a desire for more time explicitly dedicated to educational activities.

“General frustration of probably many people will be that no specific time is set aside (for both parties)” (Supervisor1, surgical specialty)

“Depending on location, the resident is still often seen as a cheap labor force that has to work a lot, rather than a colleague you train and invest in” (Resident2, other specialty)

“Doing (part of) an operation yourself, under immediate supervision with immediate feedback, is extremely rare as it would be too much of a waste of time..." (Resident3, surgical specialty)

The lack of an educational culture was another significant problem identified by the participants. Specifically, they reported often missing a clear training structure that offered a complete range of clinical experiences throughout the different rotations. Additionally, participants mentioned the highly variable quality of supervision and educational opportunities.

“Rotations, where I learned a lot, are the rotations in which staff members shared their experience with you, explained certain tasks to you, and then systematically gave you more independence in those tasks. Rotations where you can work out projects instead of just running routine.” (Resident4, other specialty)

#### MSM curriculum

The analysis revealed several concerns related to the added value of the MSM curriculum by residents. There were concerns regarding the frequency, location, and content of courses and skills training. Residents expressed appreciation for regularly scheduled courses, but also noted significant variation in the frequency of sessions, ranging from once a week to twice a year. Overall, residents felt that there were generally too few courses offered.

Residents also appreciated the option of virtual courses, which allowed them to review material at their own pace and convenience. Although face-to-face courses were seen as more stimulating and effective for learning, residents perceived them often as difficult to attend during working hours due to workplace distractions such as interrupting phone calls or clinical demands for which they were responsible.

Residents also commented on the content of the courses. Participants reported that courses related to daily practice were seen as relevant and immediately applicable to their work, opposed to frequent lectures on exceptional cases. However, they reported that there was too little choice given to the resident regarding the content of the courses they were required to take. Additionally, there was a perception that the courses lacked structure and that too few were taught by experts in the field.

**Table 1:** Summary of enablers and challenges during WPL in PGME

Theme	Subtheme	Enablers	Challenges
Dual learning path	Workplace learning	Being actively involved in performing various aspects of the job	Too little training time with an imbalance between working, and learning or teaching
			Not experiencing an educational culture
	MSM curriculum	Regularly organized courses	Frequency is highly variable
		Virtually available courses	Too little choice in offered courses
		Physically organized courses	Lacking structure in offered courses
		Courses compliant with daily clinical practice	Many distractions during physical courses
		Discipline-specific exam	The imbalance between courses given by peers and by experienced professionals
			Insufficient training in technical skills
			Insufficient training in transferable skills
			The MSM curriculum is perceived as separated on top of WPL
			Clearly defined training objectives not available or being unclear
			Preparation for the discipline-specific exam
Feedback	Frequency and timing	Systematic and scheduled feedback	Feedback being given far later than the learning experience or event
			No feedback is given at all
			Feedback fatigue due to high resident turnover
			Opposing perceptions between residents and supervisors about the quantity of feedback
			Opposing perceptions between residents and supervisors’ responsibility for initiating feedback.
	Quality	Mentioning points of improvement during feedback	Lacking positive enforcement
			Poor quality of feedback
	Two-way feedback	Supervisors wanting feedback about themselves	Residents finding few opportunities to provide supervisors with feedback
Learning support	Residents’ self-directed learning	Self-reflection included in assignments and ePortfolio	Self-reflection is complicated by lack of external input
		Research and self-study are useful	Little guidance with self-study
		Self-directed learning attitude is perceived as important	Insufficient time for self-study
			Self-directed learning skills are not mastered by all residents
			Supervisors are in need of information on how to provide proper guidance to master self-directed learning skills
	Supervisors’ guidance	Encouraging supervisors who ask questions, provide opportunity to safely fail with proper feedback, share clinical reasoning, share knowledge	Wide variation in perception regarding educational competencies of supervisors
		Supervisors being easily accessible	Solely receiving brief advice when asking for help
		Residents being considered as colleagues	A strict hierarchical structure between residents and supervisors
		Direct observation of residents	Insufficient opportunities for direct observation
		Progressively becoming more independent in a safe learning environment	Bearing inappropriate amount of responsibility (too much or too little)
			Lack of protected time for educational activities by supervisors
	ePortfolio support	ePortfolio stimulates learning conversations	ePortfolio mainly considered a logbook instead of a tool to support the learning process

“Occasionally there are classes by supervisors (actually too few, because these are more useful than yet another rare case presented by a fellow resident)." (Resident2, other specialty)

“I sometimes find it absurd how many credits we have to pay the university for "courses" that have no class content but require us to complete assignments or submit the number of conference hours for example.” (Resident5, medical specialty)

The tasks that were given in some courses were described as not always relevant and sometimes even useless. Residents also reported a lack of training related to technical skills, which were highly needed as time constraints on the job hindered their ability to gradually develop these skills during clinical care.

“You are supposed to learn this in the workplace but when you start as a resident [...] there is sometimes little time to learn a skill thoroughly. [...] Unfortunately, in practice you often have to be able to do it right away if you have seen it once or twice." (Resident6, medical specialty)

Residents also expressed a desire to learn transferable skills that could be applied across a range of clinical settings. These skills included clinical time management, stress management, and providing feedback to colleagues.

Another aspect of the MSM curriculum includes the competencies or training that form the basis of the curriculum. The lack of clearly defined training objectives was a challenge for both residents and supervisors. In some cases, the objectives were not provided or were unknown, and in other cases, multiple lists of objectives existed, which created confusion for residents and made it difficult for supervisors to monitor residents’ learning. This also made it challenging to work with learning goals. Both residents and supervisors often failed to follow up on learning goals. Additionally, many residents admitted to not formulating these goals, despite recognizing their usefulness.

“I was never asked to formulate goals, but I would find this particularly useful. Ideally, supervisors should also be aware of the goals you have formulated for yourself so that they can pay extra attention to this.” (Resident7, medical specialty)

Residents appreciated examinations related to their specialty; however, they felt that the theoretical preparation for these exams was inadequate.

“But the training here could provide more structure and guidance. Suddenly we are residents and good courses seem superfluous, while we still have to take exams.” (Resident6, medical specialty)

### Feedback

Feedback includes formative and summative feedback because participants made no difference between both feedback formats.

#### Frequency and timing

When analyzing the frequency and timing of feedback, only a minority of residents indicated that feedback was given systematically or scheduled. Regarding timing, several residents indicated that feedback was provided during or just after the learning event, but the majority stated that feedback was delayed or not provided at all.

“Feedback is often written afterward via [the current ePortfolio]. But often comes a while later, making it not as relevant.” (Resident8, medical specialty)

“But for the first 2 years of my training, it was hoping you did it right and trying to look up a lot in the literature when in doubt.” (Resident9, other specialty)

Supervisors indicated that a high resident turnover was associated with repeating the same feedback to different residents, which lead to induced feedback fatigue. This turnover also hindered feedback on the process of continuous development of competencies over time.

In terms of quantity and responsibility for initiating feedback, there was a mismatch in perceptions between residents and supervisors. Some supervisors felt that they provided a lot of feedback, but believed that residents should take the initiative to ask for feedback. Residents, on the other hand, reported receiving too little feedback and expressed a desire for supervisors to give feedback more spontaneously or provide qualitative feedback when asked for it. The following quotes exemplify this problem:

"The resident is expected to ask for feedback himself, but this rarely happens.” (Supervisor2, medical specialty)

“We have to make up for ourselves all those evaluation forms in the ePortfolio, which are then simply validated by supervisors without a conversation.” (Resident2, other specialty)

#### Quality

The analysis revealed various issues related to the quality of feedback. Supervisors stated that many colleagues hesitated to give critique on residents and find it hard to provide constructive feedback, which left many working points undiscussed. A similar result was found in the focus groups of the residents, who felt that feedback was frequently only given if something went wrong.

Additionally, residents missed specific positive enforcement as this would provide them with a clearer image of their performances. Most residents reported a low quality of feedback they received. They found it superficial and lacking in added value for their self-reflection. However, residents acknowledged that the quality of feedback was highly variable among supervisors and praised those supervisors who took the time to provide high-quality feedback.

“I really hate these feedback moments where they just say 'it was good'.” (Resident10, medical specialty)

“I spent at least an hour once […] with a fantastic supervisor who gave me a super comprehensive evaluation. He had really observed and assessed me. Cited points I knew about myself, but also brought new insights. But that takes time...” (Resident11, medical specialty)

“Evaluation moments are rated as a chore, little time is spent on this unless negative, and one ‘has to’ say something about it. Compliments are seldom given in the workplace, much is taken for granted.” (Resident12, surgical specialty)

#### Two-way feedback

Several supervisors believed that receiving feedback from residents would be beneficial. However, only a small number of residents reported being involved in a two-way feedback process. Some residents reported that the current feedback culture did not provide the opportunity to do so, or felt that feedback towards supervisors was not appreciated.

“There should also be more feedback from the resident on your performance as an educator.” (Supervisor2, medical specialty)

### Supporting the learning process

The participants described different actors and tools playing a role in supporting the learning process.

#### Residents’ self-directed learning process

Residents reported that they mostly engaged in self-assessment when it was assigned as a task or in preparation for a formal assessment. However, in daily practice, many residents struggled with adequate self-assessments as they lacked feedback from others on their performances and tended to compare themselves with their peers instead.

“I’ve become more confident in what I’m doing, but I have no idea if I’m doing it right. It has happened several times before that I do something in the way that I think I am doing it correctly, only to be told by accident (for example, as a comment to someone else) that something should actually be done differently.” (Resident9, other specialty)

Engaging in research and self-study are important aspects of the expected self-directed learning attitude of residents. While residents are motivated to take an active role in their learning, they sometimes felt overwhelmed by the vast amount of available literature. Despite seeking urgently needed case-related information during clinical encounters, they may struggle to perform a critical appraisal and conduct deeper research due to time constraints imposed by the clinic’s pressures.

“The range of study materials is so vast that as a resident it is not always easy to know where to start.” (Resident6, medical specialty)

“At work itself, little effective time is sometimes provided for e.g. scientific work, looking things up, making Master’s degree related tasks. The 60 hours that you are supposed to work weekly are often occupied with your patients, the many administrative tasks that come along with it, etc.” (Resident13, medical specialty)

While many residents acknowledged the importance of having a self-directed learning attitude, there is still room for improvement in their ability to master self-directed learning skills and supervisors also needed more information on how they could guide residents in acquiring these skills:

“Many [residents] have not mastered self-directed learning at the start [of their residency], and info and guidance for supervisors and residents is desirable.” (Supervisor3, medical specialty)

#### Supervisors’ guidance

Residents described their most valuable supervisors as those who fostered learning by asking questions, providing a safe environment for patients and residents if residents make mistakes, and offering adequate follow-up and feedback, while thinking together with the resident. They also appreciated supervisors who explained their clinical reasoning during patient care. Although supervisors were in most cases easily accessible by phone, they mainly provided only brief advice without additional information. Residents highly valued supervisors with strong teaching skills and motivation to guide residents, but perceived important differences between supervisors.

“In other places, you are left more to your own devices and you work mainly independently with short telephone consultations. You will have to do more research on your own to refine your knowledge, but this is less efficient.” (Resident14, medical specialty)

Residents felt that equality and connectedness in their relationship with supervisors empowered their learning process, whereas a hierarchical structure had an inhibiting effect:

“Others rely more on the hierarchy and then you almost don't dare to approach them.” (Resident2, other specialty)

Scaffolding the learning process was challenging as both supervisors and residents acknowledged the importance of direct observation to provide qualitative feedback, but there were limited opportunities for the supervisor to be present at the patient encounter. Many residents mentioned gradually

being entrusted with increasing responsibilities over time, but others experienced the opposite and were overwhelmed with responsibilities that exceeded their capabilities.

 “We often just accompany the supervisor and stand around watching.” (Resident14, medical specialty)

“While you are still basically just "tagging along" during your last days as an intern, a month after graduation you are dropped into a hospital and have to do a 24-hour on-call shift in emergency rooms on day 4. From no responsibility to lots of responsibility, without a decent transition period.” (Resident15, medical specialty)

The supervisors indicated that they struggled to combine all their different tasks with their teaching responsibilities. Again, one major reason for this was a lack of protected time to supervise a large number of residents.

“Maybe the function [of supervisor] should be much more untwined from clinical duties and other staff members can be involved. This is difficult because we have a lot of residents, and good supervision takes a lot of time.” (Supervisor4, medical specialty)

#### ePortfolio support

The role of the currently used ePortfolio elicited mixed feelings among residents and supervisors. While the majority of residents perceived it as merely a logbook, lacking in support for their learning process, some recognized its potential to facilitate educational conversations and foster deep self-reflection. However, these individuals felt that the ePortfolio was not being utilized to its fullest extent.

“I also find that on that front, [the current ePortfolio] sometimes feels more like an administrative task that you have to add to get your recognition rather than a real learning platform or a tool where you can track your own goals and progress.” (Resident16, surgical specialty)

Supervisors held mixed opinions on the role of the ePortfolio in supporting the long-term follow-up of residents as documented in the following quote:

“[The ePortfolio] allows logging what a resident has seen/experienced, to what extent he/she is considered competent on the different rotations where he/she has worked. Nevertheless, it remains difficult to deduce from [the ePortfolio] whether the training goals have all been met, those goals can certainly be formulated even more concretely and completely.” (Supervisor3, medical specialty)

## Discussion

This study aimed to identify the main enablers and challenges of WPL for residents and their supervisors involved in training hospital specialists across different medical specialties and clinical learning departments. Our results, summarized in Table 1, highlight various areas that can inform the development of WPL programs.

The first theme of our study focuses on the dual learning path, which consists of WPL and formal teaching sessions. While not all countries offer a Master of Specialist Medicine (MSM), postgraduate medical education usually involves a combination of WPL and formal lessons. Our results indicate that both entities currently have their value, but there is still room for improvement in balancing working and learning. Time constraints are often cited as a challenge,^7^^, ^^20^ as participants reported frustration regarding non-educational patient care administrative tasks and the lack of protected time for structured formal learning.^21^ The focus groups revealed that there is often insufficient time available at the workplace to dedicate to education, which is reflected in several themes. While supervisors and residents can optimize their available time, the implementation of their clinical time is beyond their control. Obligations, organization, and budgeting have an important influence on the time use of both parties during working hours and it is essential to consider education in organizational structures.^22^^, ^^23^

According to our results, training objectives were not being utilized effectively, and many residents reported that the existing training objectives were not sufficiently clear, which was felt to have a great impact on WPL as reflected in the existing literature.^7^^, ^^24^ In addition to defining discipline-specific competencies as training objectives, there is a growing need to foster the development of transferable skills, which are increasingly important for physicians in the current medical landscape.^25^

To optimize the PGME experience, it is important to align and complement the training objectives of WPL and formal lessons as they complement each other's deficiencies. Our study highlights that these elements are currently perceived as separate entities and suggests that the formal lessons would benefit from a clear structure. This would enable residents to construct their program based on their individual learning needs and availabilities, which vary in the unpredictable WPL context with an ill-defined curriculum.^12^ To further align WPL and formal courses, there should be a balance between face-to-face and virtual courses.

A second theme in our results was feedback. We found several challenges regarding the frequency, timing, and divergent perspectives between residents and supervisors. Although the theory of providing effective feedback is well-known, it still requires significant attention. To address this issue, a broader foundation for an adapted feedback culture needs to be established and feedback initiatives should become a shared responsibility.^26^^, ^^27^

The quality of the feedback process was another point of discussion. It is unclear whether feedback quality was indeed insufficient, or whether this was solely the perception of residents. Similar results have been found in other studies as well. ^14^ Another issue was positive reinforcement. Although much research suggests that feedback during WPL is too often only positive, our respondents stated they missed positive reinforcement.^13^^, ^^28^ This reinforcement has been shown to increase confidence in residents, stimulates them to seek more feedback, and fosters a more productive learning environment.^29^ The residents in our study indicated that positive reinforcement needs to be specific, as a general "well done" is deemed insufficient to effectively identify and maintain desirable behaviors.

Delivering high-quality feedback indeed requires providing specific cues, which in turn requires direct observation of residents.^13^ However, our findings suggest that direct observation may not always be feasible or implemented. Video observation could be an opportunity here, as the supervisor no longer needs to be present at the place and time of the residents’ clinical work.^30^ Similarly, there is minimal interference from the supervisor, who can see the resident at work in a natural situation.^31^ In addition, the residents can also observe themselves afterward, which can make the feedback dialogue more constructive.^30^^, ^^32^^-^^34^

The topic of two-way feedback arose spontaneously in all focus group discussions. Although supervisors expressed the need for feedback towards them, residents felt that it was rarely valued when they provided supervisors with feedback. The literature indicates that creating a safe environment for both residents and supervisors is crucial for hierarchically upward feedback to be effective.^35^ However, as the current feedback culture still needs improvement, this safe environment may not always be present. Standardized questionnaires might provide a way to introduce upward feedback and allow residents to practice their feedback skills.^35^^, ^^36^

The third and last theme was learning support. Residents need to develop their self-directed learning skills to support their learning. These skills are essential to achieve the required competencies in complex and unstructured learning environments, such as the clinical workplace.^21^^, ^^37^^, ^^38^ However, it appears that residents have not yet sufficiently mastered these self-directed skills or that the needed prerequisites, such as time and support, are unavailable. Although supervisors must support self-directed learning through scaffolding, supervisors indicated they need more guidance in how to do so.^39^^, ^^40^

Residents report a wide variation in perception regarding the educational competencies of supervisors. A good clinical supervisor embodies several characteristics. While it is important for supervisors to possess strong clinical skills and knowledge in their specialized area, they should not limit their role to simply transmitting information. Instead, they should strive to facilitate learning, for example, by coaching residents and entrusting them with clinical tasks that are within their competency.^41^^-^^43^ These competencies can be further developed through train-the-trainer sessions. As previously reported, two-way feedback can also support competency development in supervisors.

The relationship between supervisors and residents also has a significant impact on learning, but it is often complicated by factors such as high workload, varying levels of motivation for direct supervision, and hierarchical structures. A good working relationship between supervisors and residents is crucial for ensuring optimal patient care and optimal learning in a safe learning environment, and it should receive more attention.^44^^, ^^45^

The need for a safe learning environment was also reflected in the perceived inappropriate amount of responsibility and autonomy given to residents. This should be increasingly awarded,but residents reported that the level of responsibility and autonomy was often either too low or too high.^46^^, ^^47^ Although the literature mainly reports too little autonomy at the end of the training, the participants in these focus groups suggested that this imbalance between residents’ competency and autonomy level was already present at the beginning of their training, where they are granted too much autonomy.^48^ In contrast, they were often granted insufficient autonomy at the end. This imbalance could lead to dangerous situations, as residents may not always seek supervision in critical clinical situations, or they might feel insufficiently challenged and lose their motivation.

Currently, the perceived added value from the ePortfolio in terms of learning and supervision was rather limited. However, ePortfolios have already been shown to have possible benefits for self-directed learning and competency-based learning.^40^^, ^^49^ By providing comprehensive information regarding the residents’ current abilities, supervisors could also assign adequate responsibility in clinical practice, which is currently discussed as being a major issue in these focus groups.

This study can offer guidance to all stakeholders involved with WPL to develop a better understanding of how WPL can be optimized to improve the PGME experience. Ultimately, this research has the potential to inform the development of WPL programs that can help to improve the quality of patient care provided by medical professionals. Future studies could focus on confirming the results of this study in a broader, perhaps international setting and exploring strategies for aligning residency training programs to improve quality. Moreover, future studies could explore potential strategies for enhancing collaboration between different residency programs and institutions across Europe to facilitate the sharing of best practices and standardization of training. Further research could also be conducted on the integration of new technologies and innovative teaching methods in residency training, along with their potential enablers and challenges.

### Limitations   

The strength of this study consists of the in-depth virtual focus groups, allowing iterative responses from participants,with time for reflection as the focus groups evolved over several weeks. Participants covered a wide variety of hospital specialisms. It is the first qualitative study to include residents of different specialties on such a large scale in Flanders. The data were analyzed by two researchers in an iterative way using thematic content analysis, extracting all different topics. However, the study has also some limitations. First, we could not include all Flemish universities offering a PGME program because of limited time and budget. However, residents from different universities work in the same regional hospitals, and educational programs barely differ between universities. Also, the number of participants and focus groups ensured diversity. Second, online focus groups were performed due to COVID-19, which might have limited interaction between participants. Nevertheless, a multidisciplinary team of moderators stimulated participants to discuss online. Third, qualitative research is exploratory, so the results of this study cannot comment on the extent to which positive or problematic situations occur. Finally, there was a limited participation response which might have been partly caused by COVID-19 having an enormous impact on the workload of hospital staff at the time when the focus groups were executed. This might have led to a situation where only the most motivated residents and supervisors participated. This might have influenced our results. Participating supervisors might have been more interested in medical education and thus not reflect the opinions of the whole group of supervisors, and participating residents might have been more frustrated in their education, thus providing more negative results.

## Conclusions

The study aimed to identify the enablers and challenges for residents and their supervisors involved in training hospital specialists. Focus groups were conducted across different medical specialties and clinical teaching departments. This study highlighted various areas that can inform the further development of WPL programs. Three themes emerged, including dual learning paths, feedback, and learning support. This study can offer guidance to all stakeholders involved with WPL to develop a better understanding of how WPL can be optimized to improve the PGME experience. Further research is needed to confirm these results in a broader international setting and explore strategies for aligning residency training programs.

### Acknowledgments

The authors would like to acknowledge the contribution of Dr. Anissa All and Ms. Sofie Van Ostaeyen that accommodated moderating the focus groups, Ms. Helena De Mey for support and discussion during the coding process, and Mrs. Vasiliki Andreou for proofreading this manuscript.

This research was embedded in the SBO Scaffold project (A19-TT-0834 ND_FWO - SBO), which is funded by Research Foundation Flanders (FWO Strategic Basic Research (SBO) (Principal funding)). The SBO Scaffold project is a four-year interdisciplinary project to improve the quality of healthcare education in Flanders through innovative technological applications.

### Conflict of Interest

The authors declare that they have no conflict of interest.
